# Supplementary material for: Characterization and whole genome sequencing of Saccharomyces cerevisiae strains lacking several amino acid transporters: Tools for studying amino acid transport
Source: PLoS One. 2025 Apr 30;20(4):e0315789. doi: 10.1371/journal.pone.0315789 (PMC12043151; doi:10.1371/journal.pone.0315789)
Supplement: S1 Fig — Yeast cells were grown overnight in synthetic defined (SD) medium supplemented with uracil. OD for each strain was adjusted to 0.1, 0.01 and 0.001. Drops of 4 µ L were aligned on minimum medium containing labeled amino acid at 3 (a) or 12 mmol/l-1 (b) as sole nitrogen source. Pictures were taken after 2.5 days growth at 30˚C. (PDF) [file pone.0315789.s001.pdf]

(a)

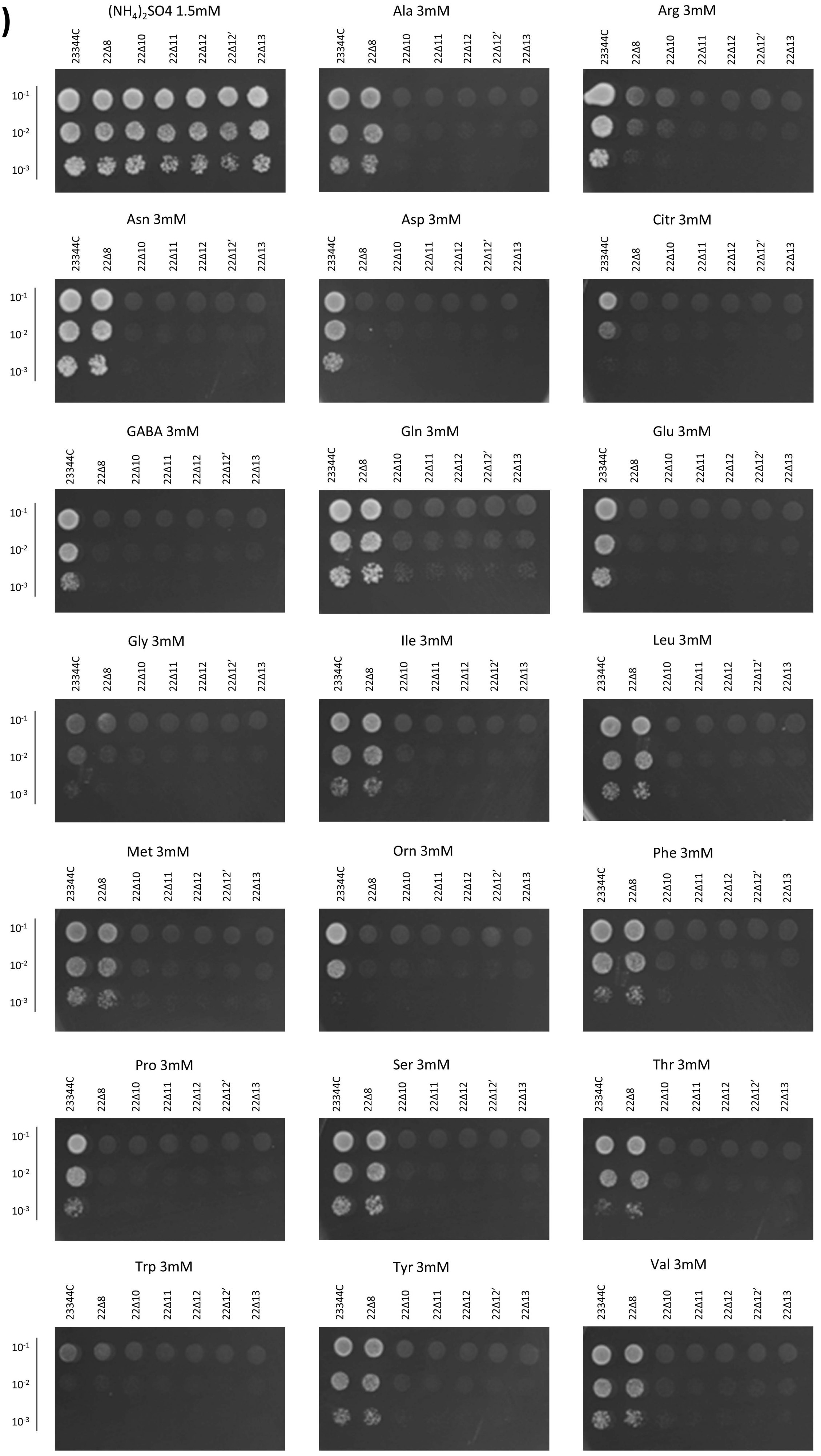

$(NH_4)_2SO4$ 1.5mM
Ala 3mM
Arg 3mM
Asn 3mM
Asp 3mM
Citr 3mM
GABA 3mM
Gln 3mM
Glu 3mM
Gly 3mM
Ile 3mM
Leu 3mM
Met 3mM
Orn 3mM
Phe 3mM
Pro 3mM
Ser 3mM
Thr 3mM
Trp 3mM
Tyr 3mM
Val 3mM
23344C
22Δ8
22Δ10
22Δ11
22Δ12
22Δ12’
22Δ13
10^{-1}
10^{-2}
10^{-3}

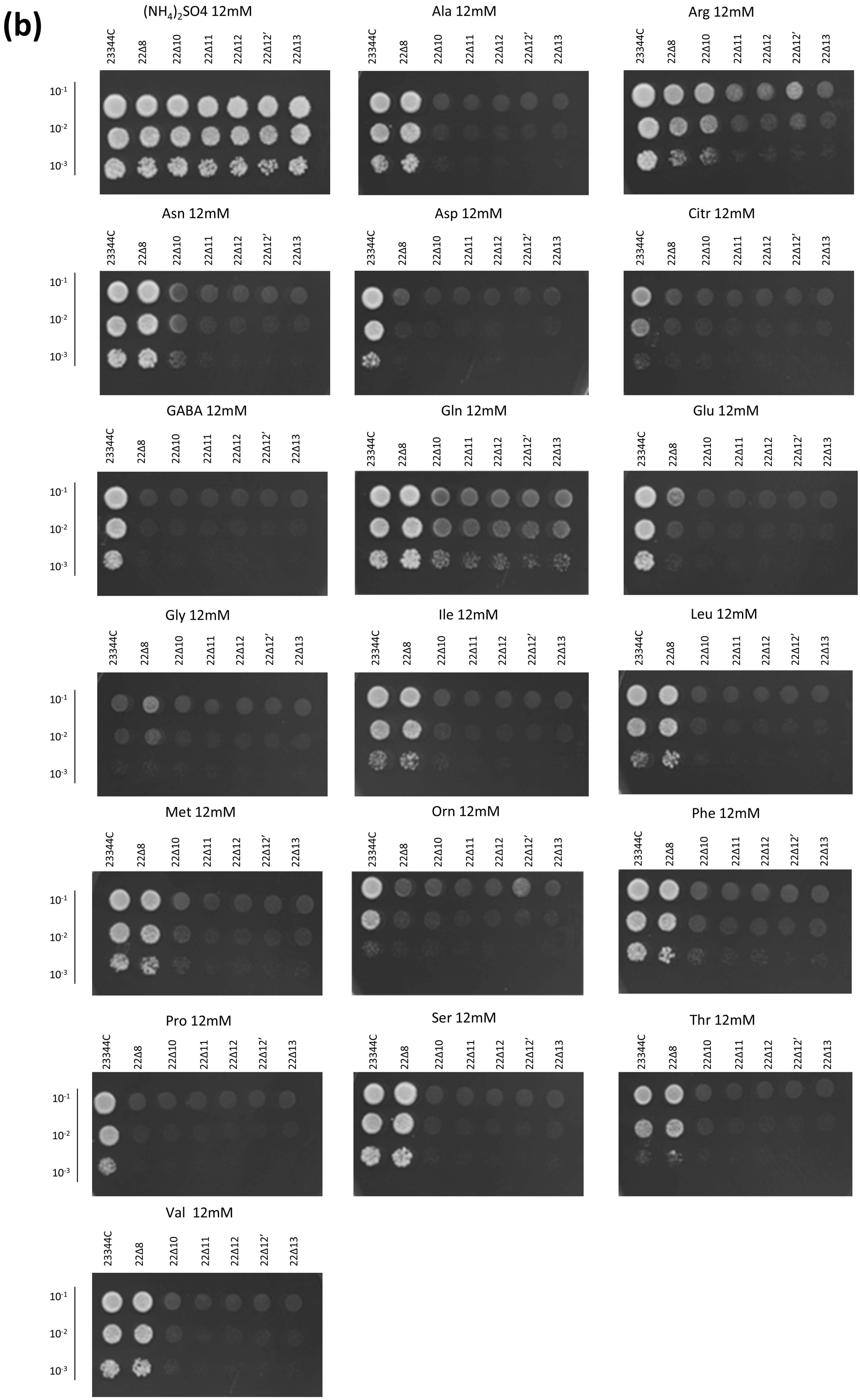
(b)
$(NH_4)_2SO4$ 12mM
Ala 12mM
Arg 12mM
Asn 12mM
Asp 12mM
Citr 12mM
GABA 12mM
Gln 12mM
Glu 12mM
Gly 12mM
Ile 12mM
Leu 12mM
Met 12mM
Orn 12mM
Phe 12mM
Pro 12mM
Ser 12mM
Thr 12mM
Val 12mM
23344C
22Δ8
22Δ10
22Δ11
22Δ12
22Δ12'
22Δ13
10^{-1}
10^{-2}
10^{-3}

S1 Fig. Growth assay comparing growth of 23344C, 22Δ10α, 22Δ11, 22Δ12, 22Δ12' and 22Δ13 cells on given amino acid as sole nitrogen source. Yeast cells were grown overnight in synthetic defined (SD) medium supplemented with uracil. OD for each strain was adjusted to 0.1, 0.01 and 0.001. Drops of 4µL were aligned on minimum medium containing labeled amino acid at 3 (**a**) or 12 $mmol.l^{-1}$ (**b**) as sole nitrogen source. Pictures were taken after 2.5 days growth at 30˚C.
