# Supplementary material for: Characterization and whole genome sequencing of Saccharomyces cerevisiae strains lacking several amino acid transporters: Tools for studying amino acid transport
Source: PLoS One. 2025 Apr 30;20(4):e0315789. doi: 10.1371/journal.pone.0315789 (PMC12043151; doi:10.1371/journal.pone.0315789)
Supplement: S2 Fig — 22 ∆ 10α, 22 ∆ 11, 22 ∆ 12’, and 22 ∆ 13 yeast strains were transformed with empty vector pRS-Ws or pRS-Ws vector containing the cDNA of GAP1 (General Amino acid Permease I, YKR039W). Yeast cells were grown overnight in selective medium. OD for each culture was adjusted to 1, 0.1 and 0.01. Drops of 5 µ L were aligned on minimum medium containing amino acids at 0.5, 3, 9 or 12 mmol/l as sole nitrogen source, and grown at 30˚C. Picture shown were taken after the number of days indicated in the table. For each amino acid/ concentration combination, the dilutions of cells were dropped on a single Petri dish, despite them being shown as strips. (PDF) [file pone.0315789.s002.pdf]

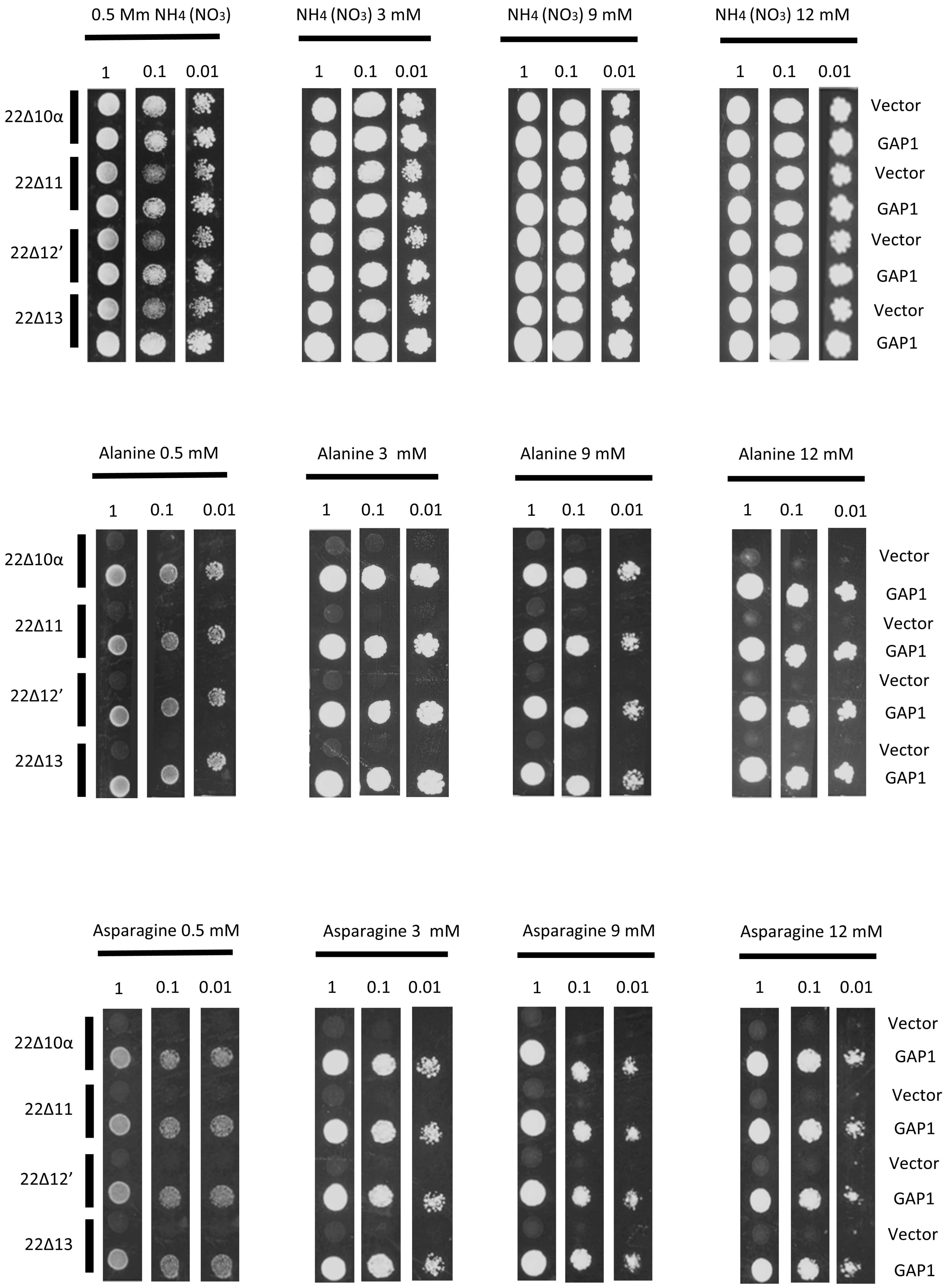

0.5 Mm NH4 (NO3)
NH4 (NO3) 3 mM
NH4 (NO3) 9 mM
NH4 (NO3) 12 mM
1 0.1 0.01
22Δ10α
22Δ11
22Δ12’
22Δ13
Vector
GAP1
Vector
GAP1
Vector
GAP1
Vector
GAP1
Alanine 0.5 mM
Alanine 3 mM
Alanine 9 mM
Alanine 12 mM
1 0.1 0.01
22Δ10α
22Δ11
22Δ12’
22Δ13
Vector
GAP1
Vector
GAP1
Vector
GAP1
Vector
GAP1
Asparagine 0.5 mM
Asparagine 3 mM
Asparagine 9 mM
Asparagine 12 mM
1 0.1 0.01
22Δ10α
22Δ11
22Δ12’
22Δ13
Vector
GAP1
Vector
GAP1
Vector
GAP1
Vector
GAP1

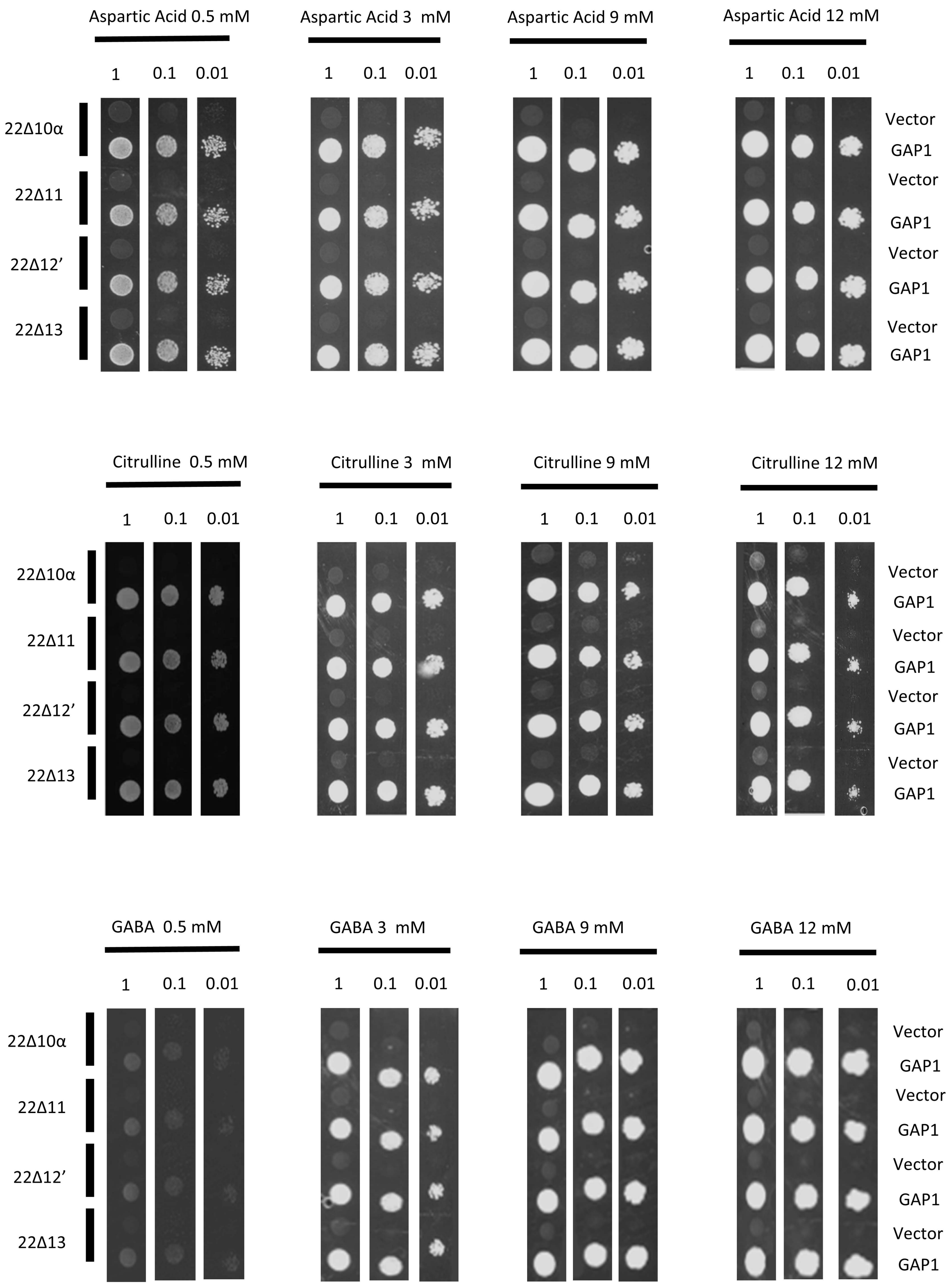

Aspartic Acid 0.5 mM
Aspartic Acid 3 mM
Aspartic Acid 9 mM
Aspartic Acid 12 mM
1 0.1 0.01
22Δ10α
22Δ11
22Δ12’
22Δ13
Vector
GAP1
Citrulline 0.5 mM
Citrulline 3 mM
Citrulline 9 mM
Citrulline 12 mM
GABA 0.5 mM
GABA 3 mM
GABA 9 mM
GABA 12 mM

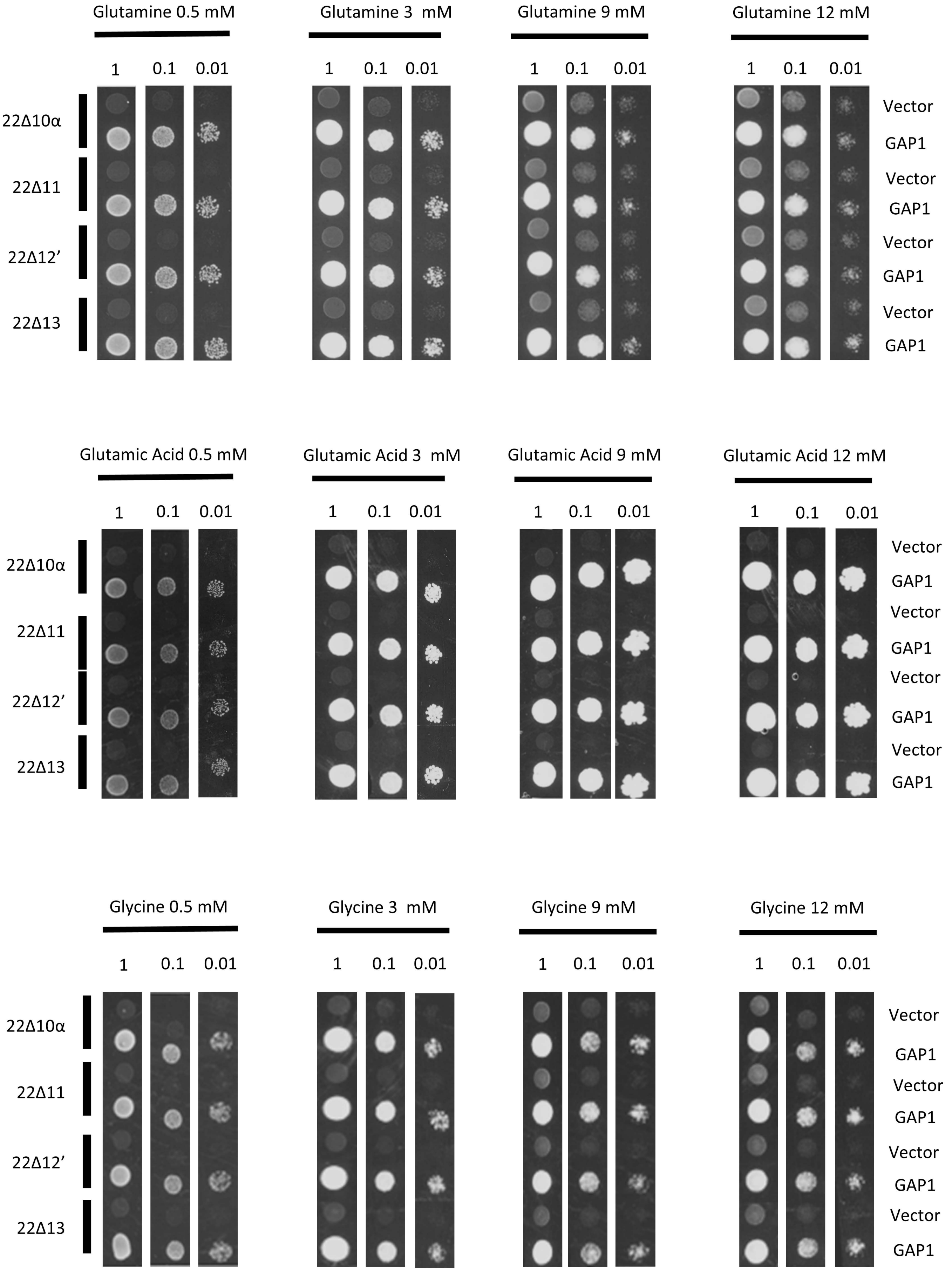
Glutamine 0.5 mM
Glutamine 3 mM
Glutamine 9 mM
Glutamine 12 mM
1
0.1
0.01
22Δ10α
22Δ11
22Δ12’
22Δ13
Vector
GAP1
Glutamic Acid 0.5 mM
Glutamic Acid 3 mM
Glutamic Acid 9 mM
Glutamic Acid 12 mM
Glycine 0.5 mM
Glycine 3 mM
Glycine 9 mM
Glycine 12 mM

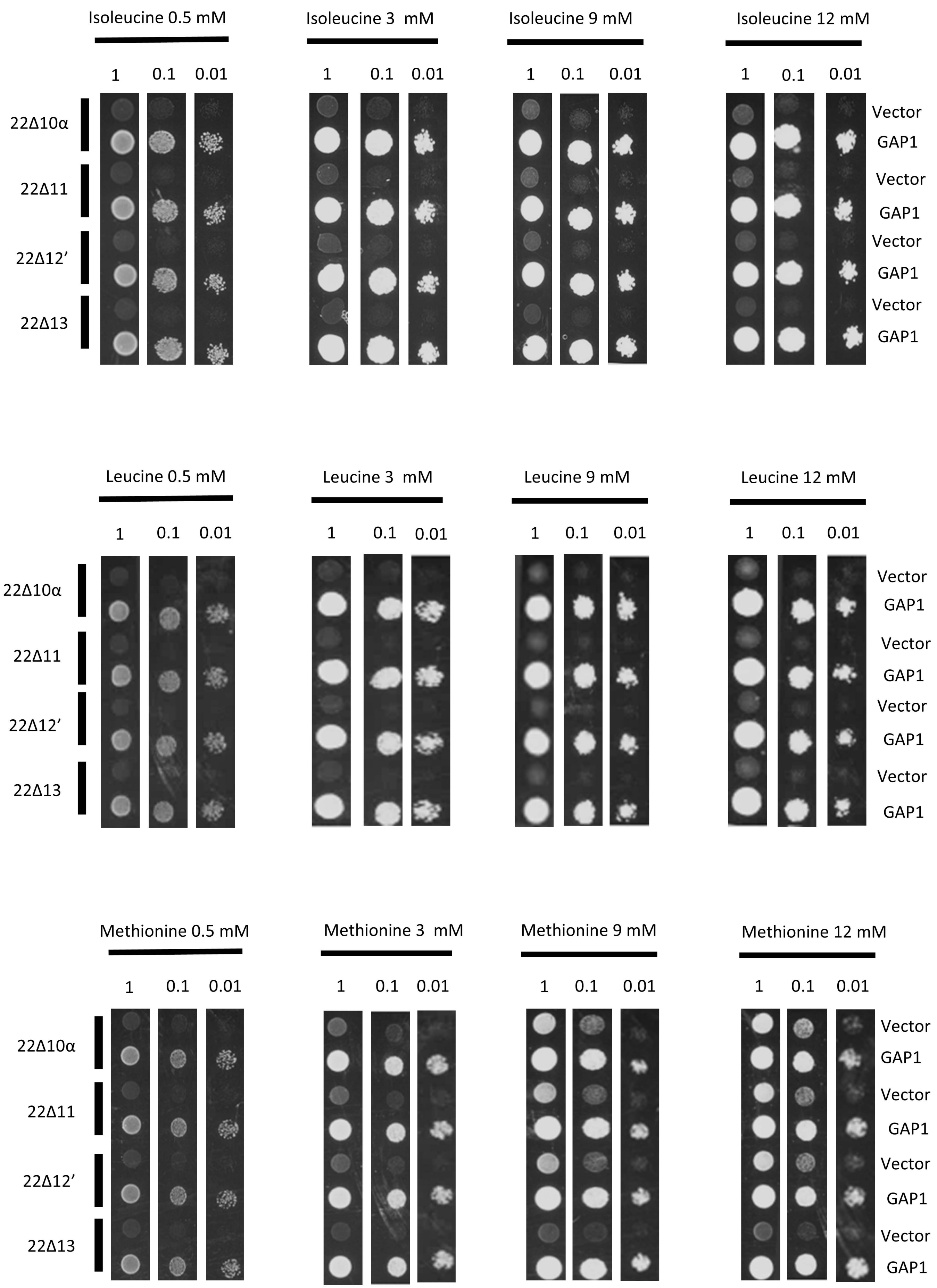
Isoleucine 0.5 mM
Isoleucine 3 mM
Isoleucine 9 mM
Isoleucine 12 mM
1
0.1
0.01
22Δ10α
22Δ11
22Δ12'
22Δ13
Vector
GAP1
Leucine 0.5 mM
Leucine 3 mM
Leucine 9 mM
Leucine 12 mM
Methionine 0.5 mM
Methionine 3 mM
Methionine 9 mM
Methionine 12 mM

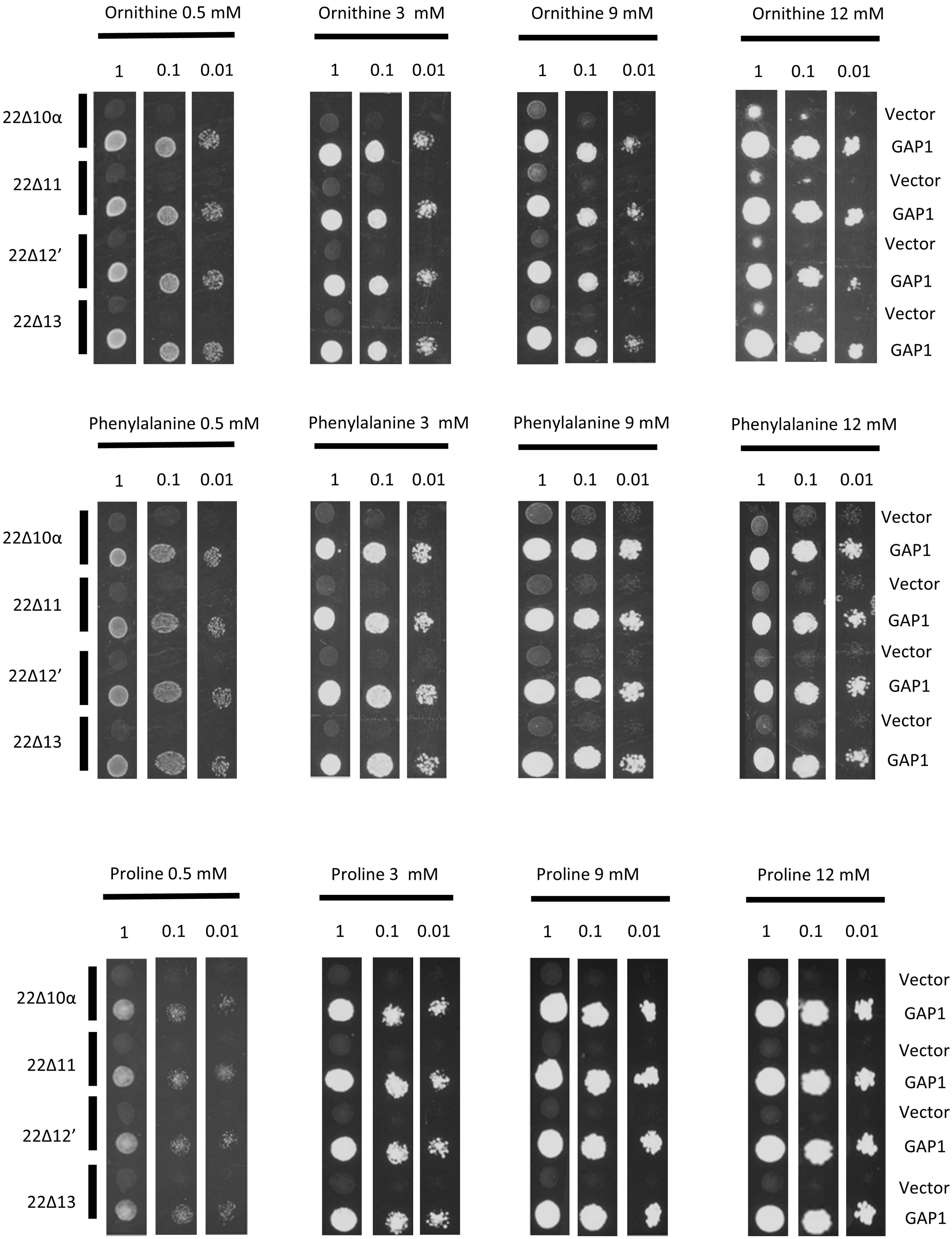
Ornithine 0.5 mM
Ornithine 3 mM
Ornithine 9 mM
Ornithine 12 mM
1
0.1
0.01
22Δ10α
22Δ11
22Δ12’
22Δ13
Vector
GAP1
Vector
GAP1
Vector
GAP1
Vector
GAP1
Phenylalanine 0.5 mM
Phenylalanine 3 mM
Phenylalanine 9 mM
Phenylalanine 12 mM
1
0.1
0.01
22Δ10α
22Δ11
22Δ12’
22Δ13
Vector
GAP1
Vector
GAP1
Vector
GAP1
Vector
GAP1
Proline 0.5 mM
Proline 3 mM
Proline 9 mM
Proline 12 mM
1
0.1
0.01
22Δ10α
22Δ11
22Δ12’
22Δ13
Vector
GAP1
Vector
GAP1
Vector
GAP1
Vector
GAP1

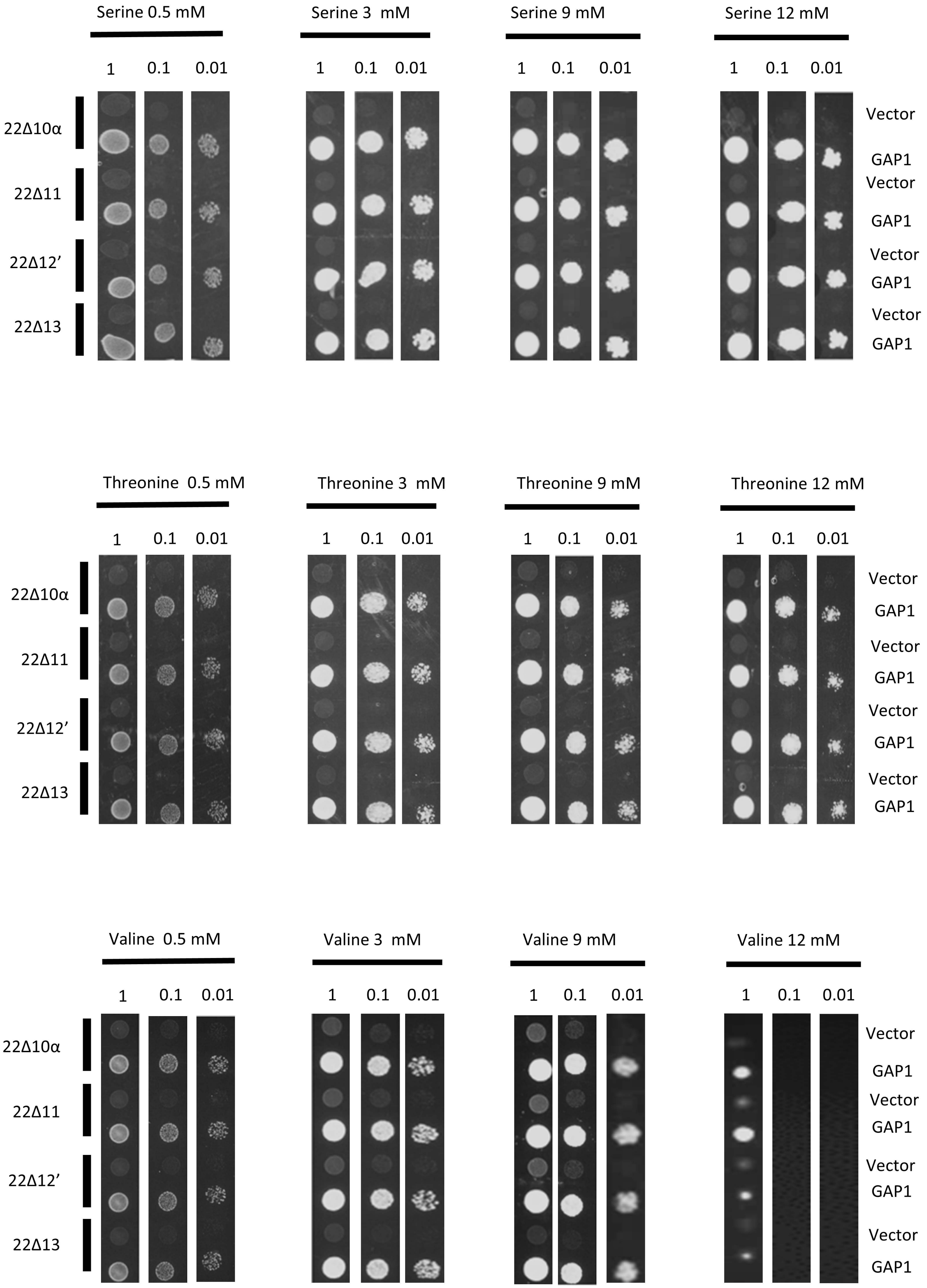

Serine 0.5 mM
Serine 3 mM
Serine 9 mM
Serine 12 mM
1
0.1
0.01
22Δ10α
22Δ11
22Δ12’
22Δ13
Vector
GAP1
Vector
GAP1
Vector
GAP1
Vector
GAP1
Threonine 0.5 mM
Threonine 3 mM
Threonine 9 mM
Threonine 12 mM
1
0.1
0.01
22Δ10α
22Δ11
22Δ12’
22Δ13
Vector
GAP1
Vector
GAP1
Vector
GAP1
Vector
GAP1
Valine 0.5 mM
Valine 3 mM
Valine 9 mM
Valine 12 mM
1
0.1
0.01
22Δ10α
22Δ11
22Δ12’
22Δ13
Vector
GAP1
Vector
GAP1
Vector
GAP1
Vector
GAP1

Days at 30°C	3	6	7	9
$(NH4)_2SO_4$			x[1]	
Ala		x		
Asn	x			
Asp	9, 12 mM	0.5, 3 mM		
Cit		x		
GABA		3, 6, 12 mM		0.5 mM
Gln	x			
Glu	x			
Gly		x		
Ile		x		
Leu			x	
Met			x	
Orn	0.5, 3, 6 mM	12 mM		
Phe	x			
Pro			x	
Ser	x			
Thr	x			
Val			x	

[1] all plates were scanned at that time

S2 Fig. Functional complementation assay of 22Δ10α, 22Δ11, 22Δ12’, and 22Δ13 yeast strains. 22Δ10α, 22Δ11, 22Δ12’, and 22Δ13 yeast strains were transformed with empty vector pRS-Ws or pRS-Ws vector containing the cDNA of GAP1 (General Amino acid Permease I, YKR039W). Yeast cells were grown overnight in selective medium. OD for each culture was adjusted to 1, 0.1 and 0.01. Drops of 5 µL were aligned on minimum medium containing amino acids at 0.5, 3, 9 or 12 mmol/l as sole nitrogen source, and grown at 30˚C. Picture shown were taken after the number of days indicated in the table. All dilutions of cells were dropped on a single Petri dish for each amino acid / concentration combination, despite them being shown as strips.
