# Supplementary material for: Characterization and whole genome sequencing of Saccharomyces cerevisiae strains lacking several amino acid transporters: Tools for studying amino acid transport
Source: PLoS One. 2025 Apr 30;20(4):e0315789. doi: 10.1371/journal.pone.0315789 (PMC12043151; doi:10.1371/journal.pone.0315789)
Supplement: S3 Fig — (a-i) 22 ∆ 10α PacBio sequencing reads were aligned to the S288C reference genome and were loaded in Integrative Genomics Viewer [35] along with the reference genome and its annotation. In mapped reads, deletions are indicated by a black line and insertions by purple regions (numbers indicated length of deletion or insertion). When a read is clipped by more than 100 bp, the end of that read is marked in red. Indels of less than 30 bp are not labeled. (PDF) [file pone.0315789.s003.pdf]

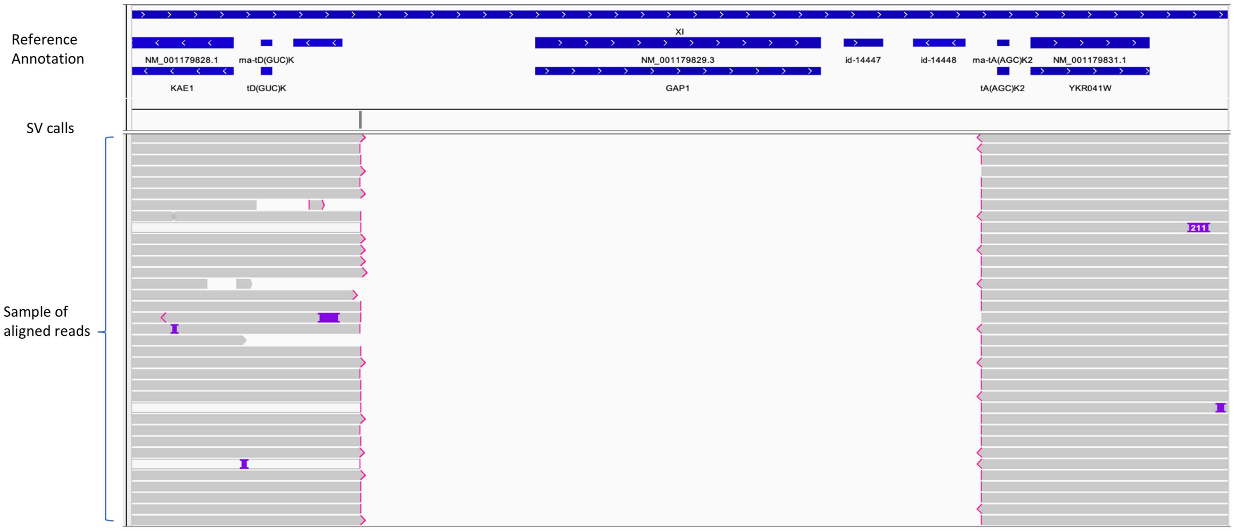

(a) GAP1

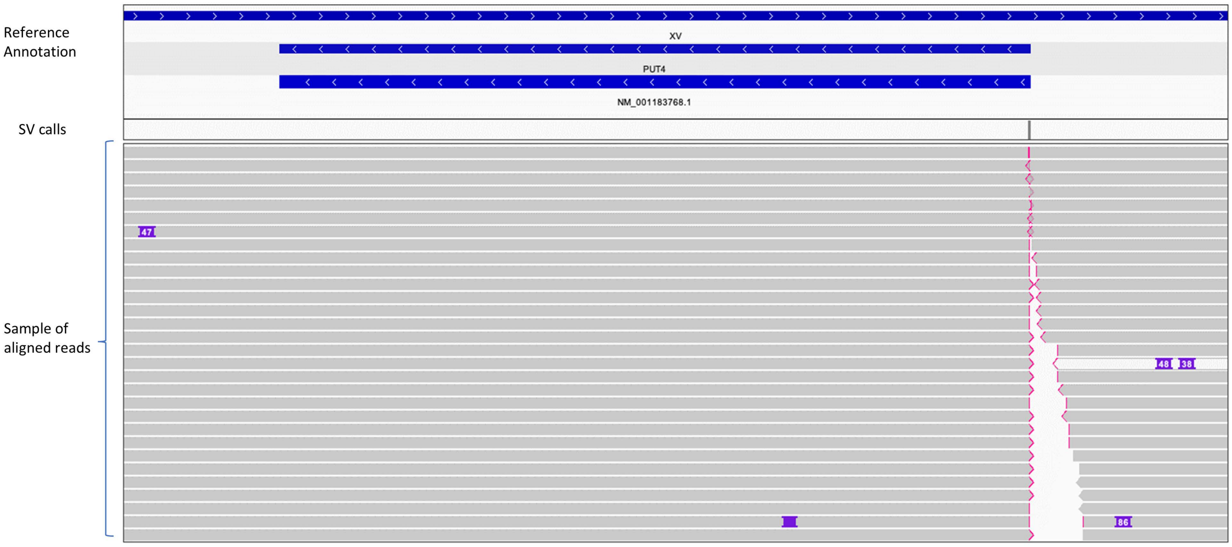

(b) PUT4

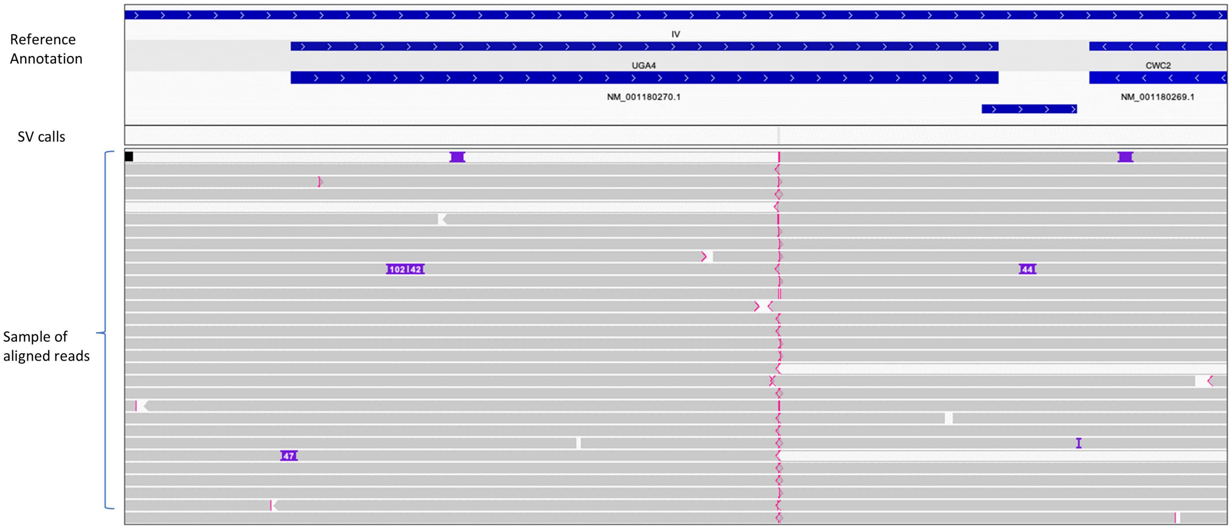

(c) UGA4

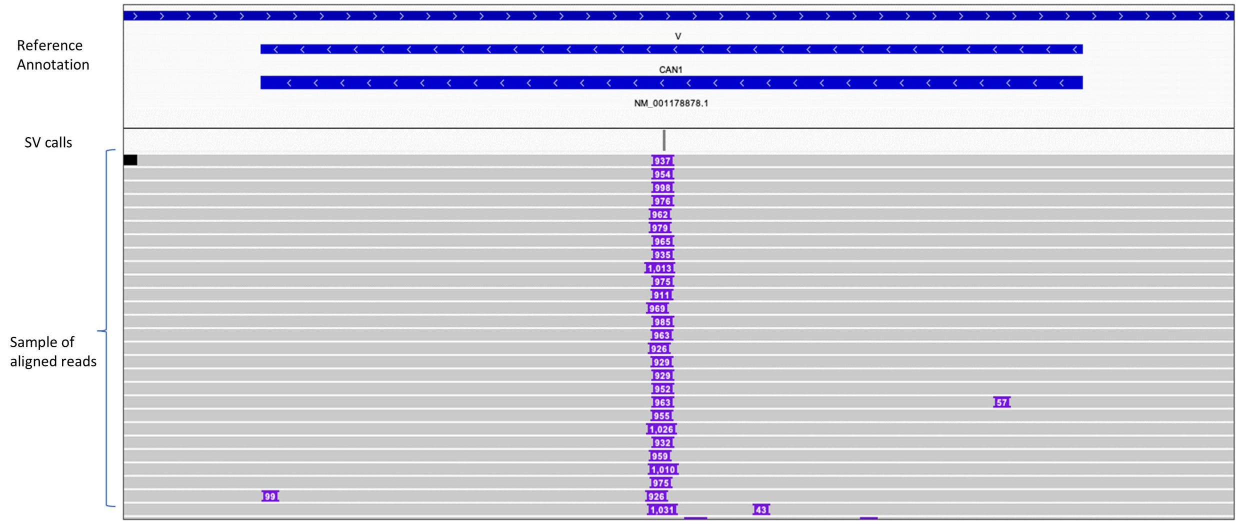

(d) CAN1

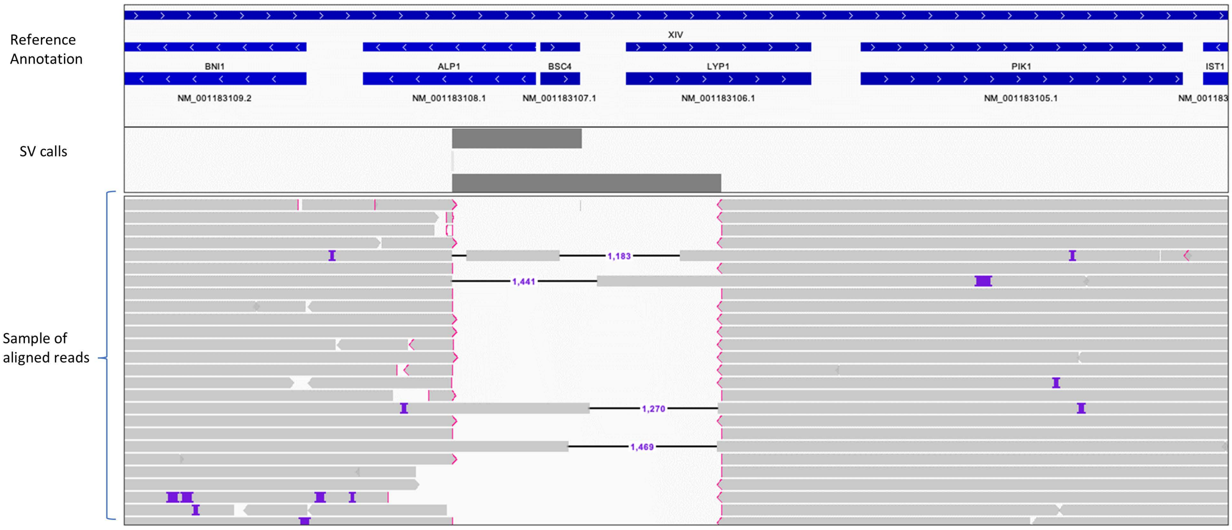

(e) ALP1-LYP1

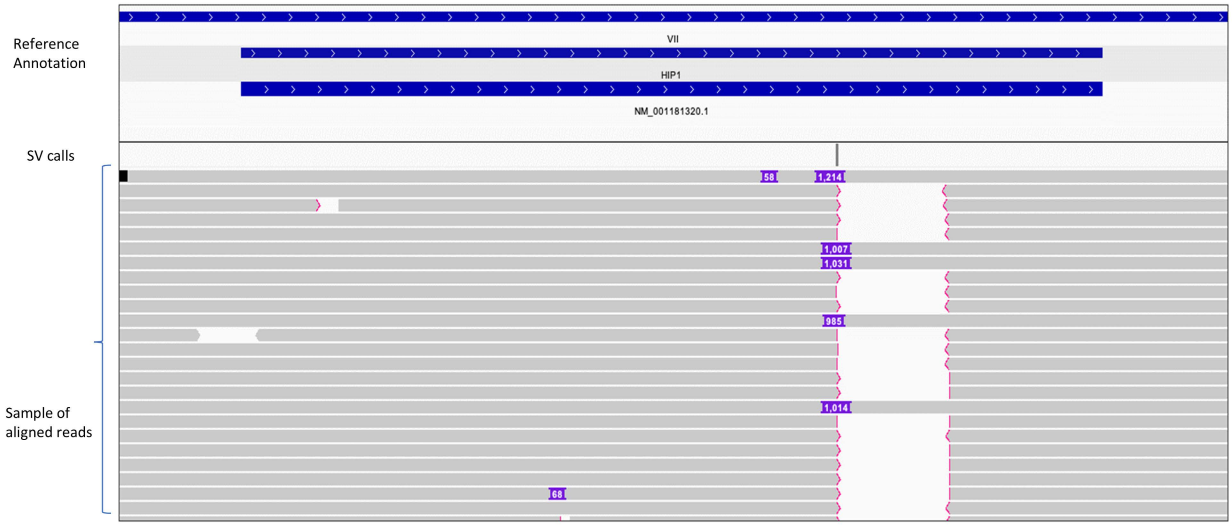

(f) HIP1

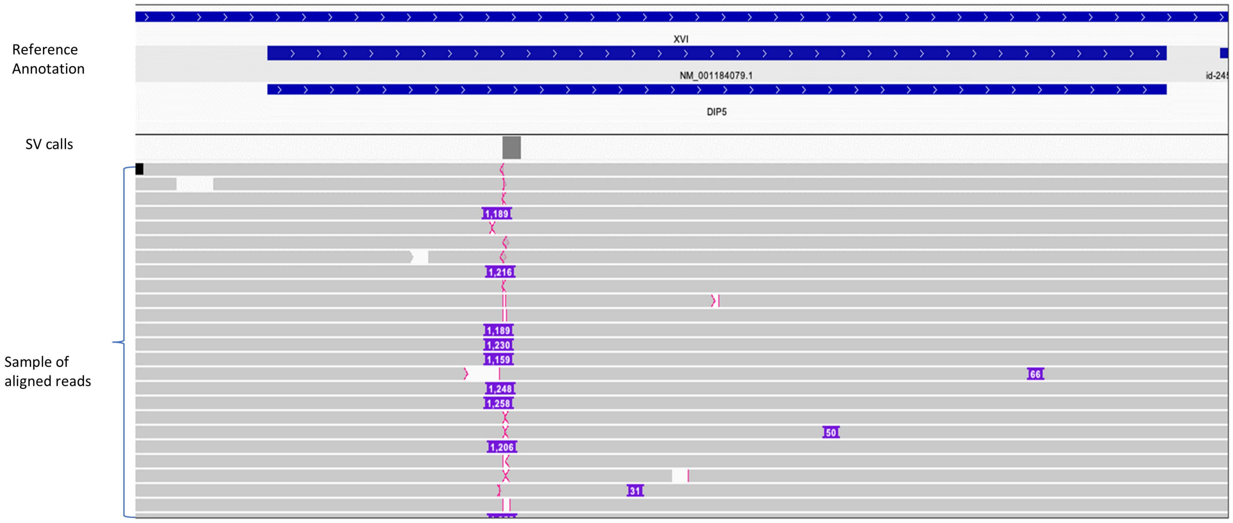

(g) DIP5

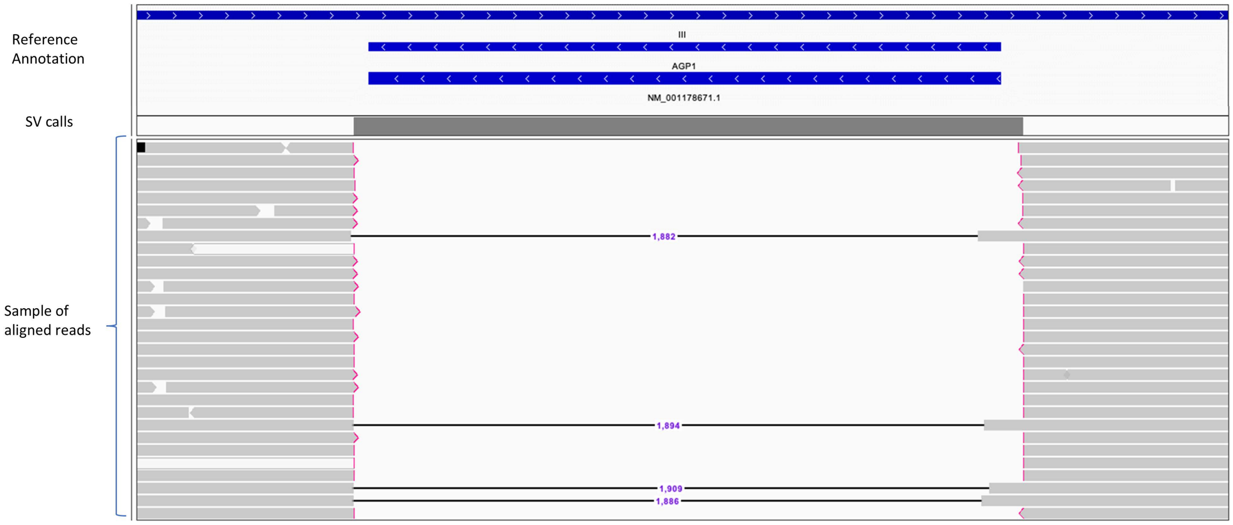

(h) AGP1

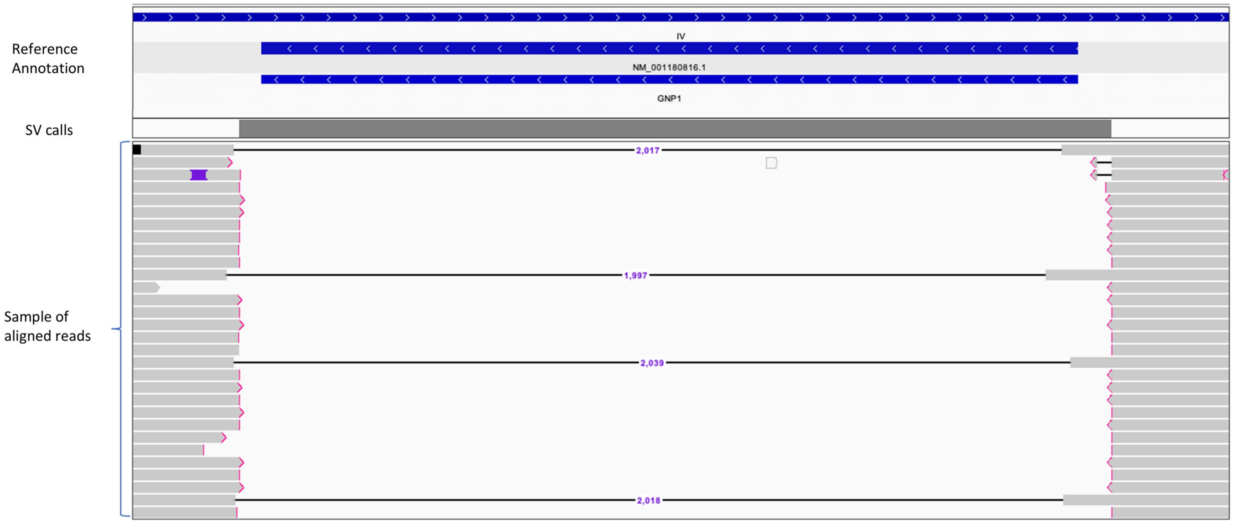

(i) GNP1

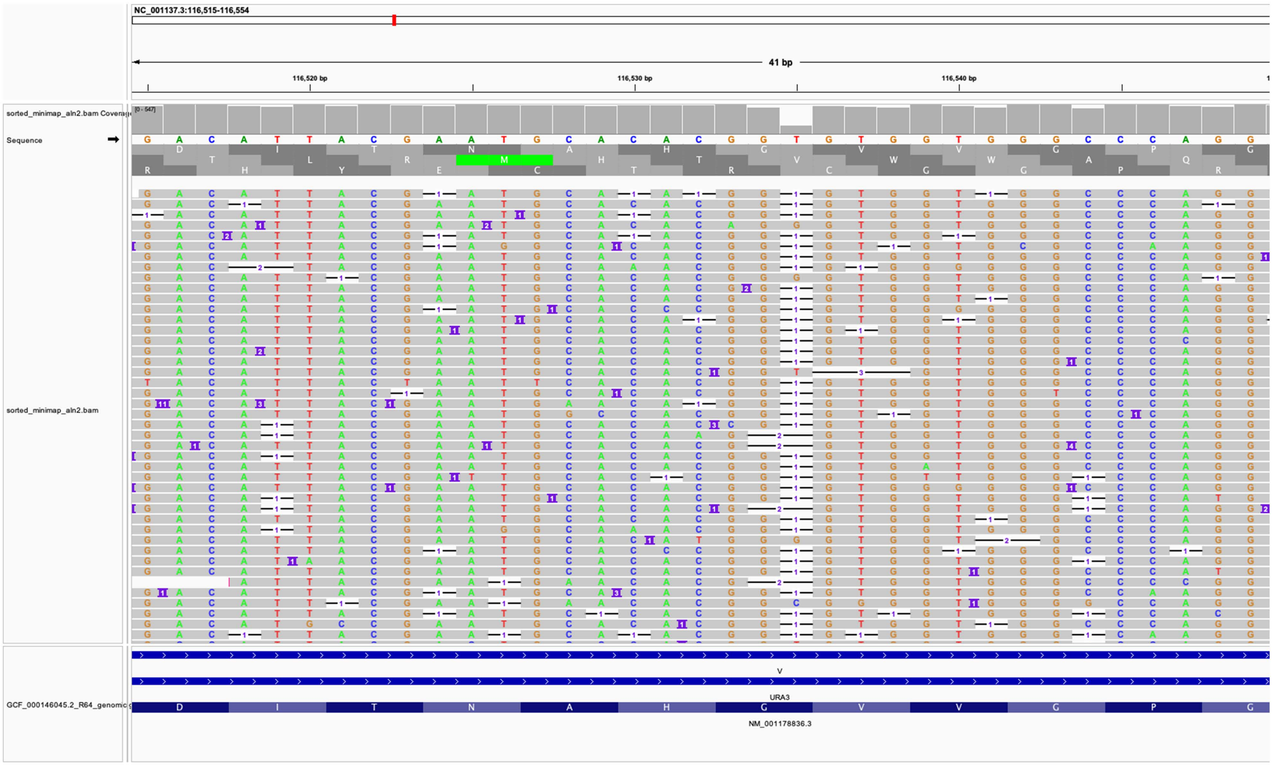

(j) URA3

S3 Fig. Integrated Genome Viewer images of indels in 22Δ10α (a-i) 22Δ10α PacBio sequencing reads were aligned to the S288C reference genome and were loaded in Integrative Genomics Viewer [35] along with the reference genome and its annotation. In mapped reads, deletions are indicated by a black line and insertions by purple regions (numbers indicated length of deletion or insertion). When a read is clipped by more than 100 bp, the end of that read is marked in red. Indels of less than 30 bp are not labeled.
