# Supplementary material for: Characterization and whole genome sequencing of Saccharomyces cerevisiae strains lacking several amino acid transporters: Tools for studying amino acid transport
Source: PLoS One. 2025 Apr 30;20(4):e0315789. doi: 10.1371/journal.pone.0315789 (PMC12043151; doi:10.1371/journal.pone.0315789)
Supplement: S4 Fig — 22 ∆ 10α assembly statistics (left); plot of the cumulative length of the assembly vs. number of scaffolds (right). (PDF) [file pone.0315789.s004.pdf]

Assembly	22∆10a final assembly
No. scaffolds	48
Total length	12874309
Largest contig	1535199
Total length	12873563
GC (%)	38.13
N50	921506
N75	684320
L50	6
L75	10

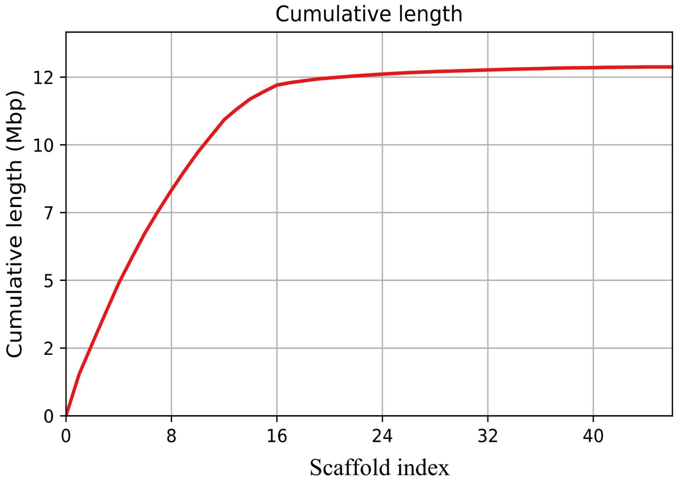

S4 Fig. Assembly statistics of 22Δ10α genome.

22Δ10α assembly statistics (left); plot of the cumulative length of the assembly vs. number of scaffolds (right)
