# Supplementary material for: Characterization and whole genome sequencing of Saccharomyces cerevisiae strains lacking several amino acid transporters: Tools for studying amino acid transport
Source: PLoS One. 2025 Apr 30;20(4):e0315789. doi: 10.1371/journal.pone.0315789 (PMC12043151; doi:10.1371/journal.pone.0315789)
Supplement: S5 Fig — Yeast strains were grown in YPDA (a) or SD supplemented with URA (b) medium, and their ODs measured using a plate reader, every 5 min for 15 hours. The data were fitted to a standard form of logistic equations to get the growth characteristics including doubling time. Each boxplot the distribution of represents six data points per strain. * p-value<0.05. (PDF) [file pone.0315789.s005.pdf]

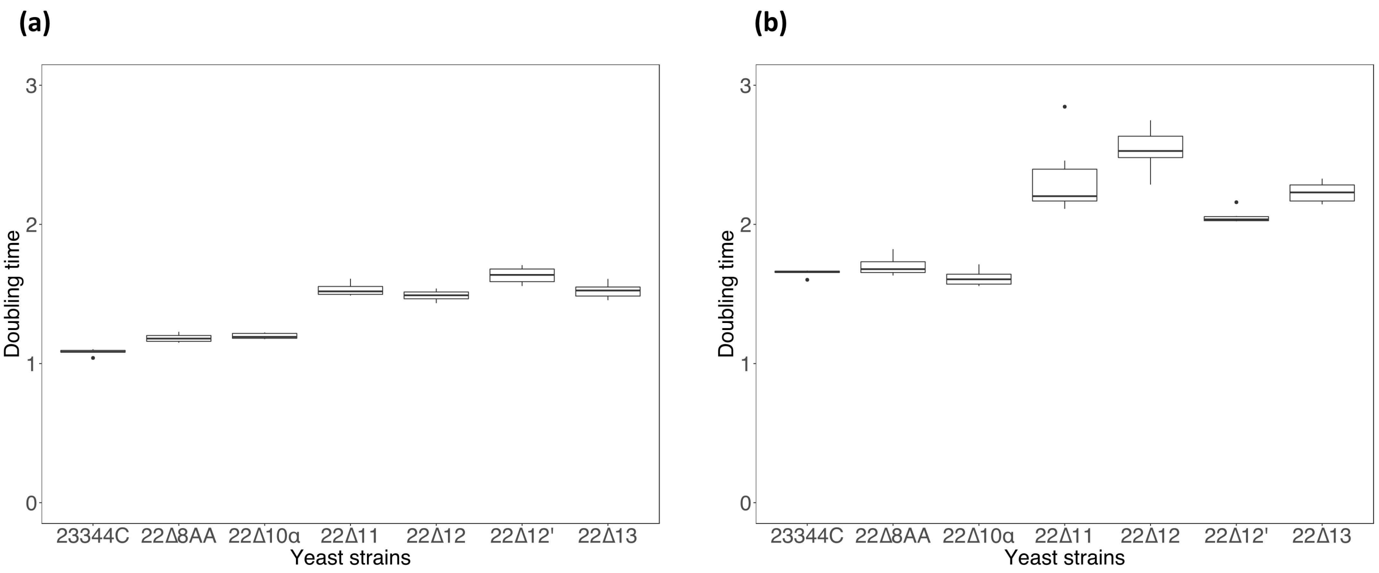

S5 Fig. Doubling time of yeast strains in YPDA and SD medium. Yeast strains were grown in YPDA (a) or SD supplemented with URA (b) medium, and their ODs measured using a plate reader, every 5 min for 15 hours. The data were fitted to a standard form of logistic equations to get the growth characteristics including doubling time. Each boxplot the distribution of represents six data points per strain. * p-value<0.05
