# Supplementary figures and images for: Characterization and whole genome sequencing of Saccharomyces cerevisiae strains lacking several amino acid transporters: Tools for studying amino acid transport
Source: PLoS One. 2025 Apr 30;20(4):e0315789. doi: 10.1371/journal.pone.0315789 (PMC12043151; doi:10.1371/journal.pone.0315789)

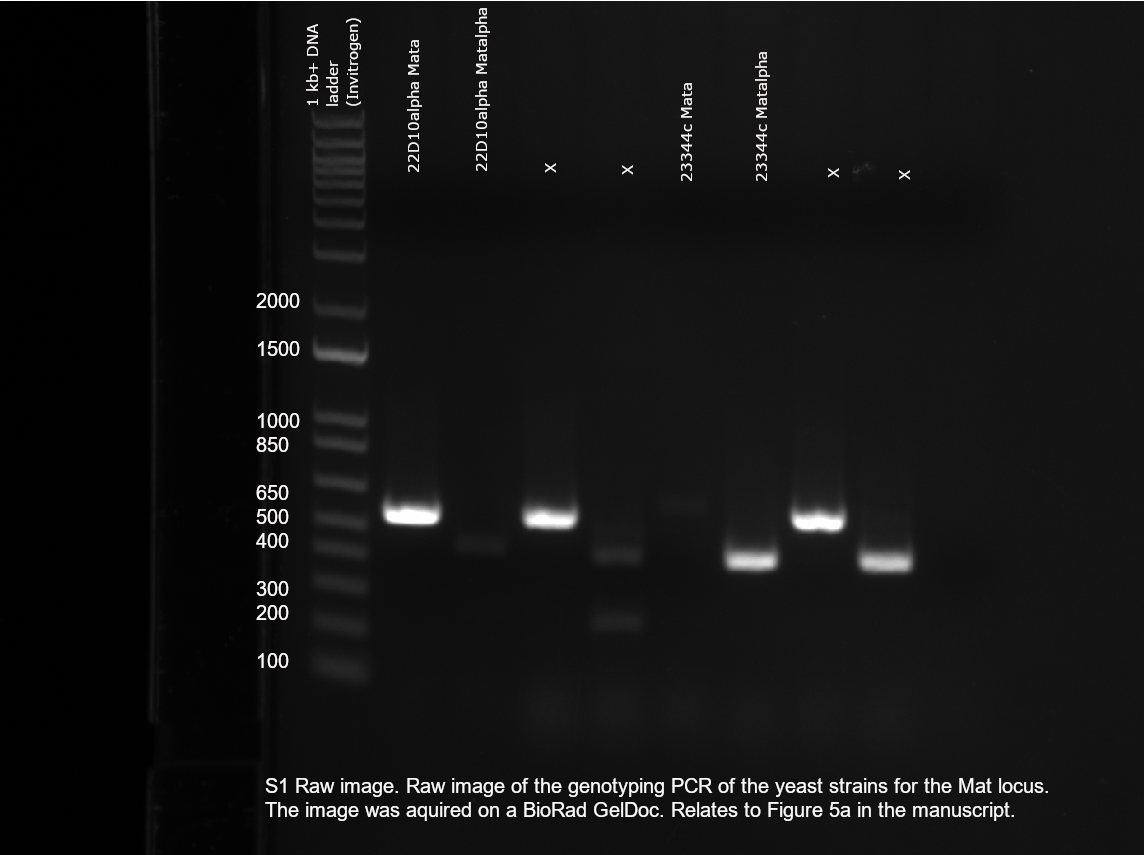

Supplement: S1 Raw Image — (TIF) [file pone.0315789.s007.tif]
