# Supplementary material for: Characterization and whole genome sequencing of Saccharomyces cerevisiae strains lacking several amino acid transporters: Tools for studying amino acid transport
Source: PLoS One. 2025 Apr 30;20(4):e0315789. doi: 10.1371/journal.pone.0315789 (PMC12043151; doi:10.1371/journal.pone.0315789)
Supplement: Attachment — Submitted filename: Response to Reviewers.docx [file pone.0315789.s009.docx]

**Reviewer #1:**

Major comments:

1. There should be a separate discussion section, not blended in with the results section. Please reformat the manuscript.

Response: Thank you for this suggestion. We have thoroughly revised the manuscript, and it now has a separate discussion that addresses the most relevant results.

2. Regarding the discrepancy between the drop test assays (figure 2) and the radioactive uptake results (figure 3), I strongly recommend the authors to run a proteomic mass spectrometry analysis in the different strains and specifically look at protein levels of the remaining amino acid permeases still expressed in the strains. This will allow the authors to know whether or not there are some compensatory mechanisms (upregulation of certain transporters?) that could explain the discrepancy.

Response: We agree that more work is needed to understand this discrepancy, and we really would have liked to address it. Unfortunately, due to limited resources and time, in addition to the fact that this falls out of the scope of this manuscript, we could not perform additional experiments (proteomics or at least transcriptomics) to explain this problem.

3. I am not sure if this is a compression problem that occurred during the submission process but please make sure to improve the resolution of all the figures (main and supplementary) if accepted for publication. As of now, the figures are very hard to read.

Response: We checked the uploaded figure files and compared it to the agglomerated pdf that was likely sent to the reviewers after processing by the PlosONE website, and we found that the original file has enough resolution and details to be legible with high magnification, while the one processed by the website has not (See Figure 1 below). We are not sure how to address this issue which is out of our control. In addition, we increased the resolution of S1 Fig, so that it is more legible. Thank you for pointing that out.

Minor comments:

1. Some sentences have grammatical errors and are hard to understand (line 80-83) ; even some words missing (line 45). Please correct.

Response: We apologize for this oversight. We carefully checked the manuscript, and those, in addition to others, should now be corrected.

2. Line 187: Could the authors comment in the text on their unsuccessful attempts to engineer additional deletions in the strain? Do they think it is due to synthetic lethality or technical problems?

Response: We added a few sentences to explain the reason we did not pursue this further: Lines 335-344 in the discussion. “We initially followed up with deleting additional genes and intended to delete *TAT2* from 22∆13 as an avenue to decrease the background growth further, but with no success. Because the kanMX cassette had not been removed from the 22∆13 genome, we used the natNT2 cassette which provides resistance to the antibiotic cloNAT [56], thereby skipping one step in the process of creating higher order mutants. *TAT2* deletion leads to viable strains, proven by the viability of 22∆12’. It is possible that our conditions were not optimal for selecting the cells which successfully inserted the cassette into *TAT2*, or that the double mutant *TAT1* *TAT2* in the 22∆11background is not viable. We did not pursue this further because the higher order deletion mutants 22∆11, 22∆12, 22∆12’ and 22∆13 did not show noticeable growth differences compared to 22∆10α on selective amino acid media (Fig 2).”

4. Line 197: Add glu to the list of amino acids in which 22Δ8AA strain cannot grow.

Response: Corrected. Thank you for noticing this.

3. For Fig S2: For the drop tests, I would have preferred to have the cells growing on the same plate for each amino acid tested instead of being patched together from three different plates. Right now, it raises suspicions as to whether or not the different dilution of cells were grown in the same conditions.

Response: We agree that the layout of this figure looks suspicious. We decided to design it that way because, for some amino acids, the rows of yeast dilutions were dropped a little far apart, which would have made a too large figure. We added the text “For each amino acid / concentration combination, the dilutions of cells were dropped on a single Petri dish, despite them being shown as strips” in the legend. See also Fig 2 below for a comparison of a panel of the figure and the original Petri dish scan. We would be happy to add the original scans in the supporting information if desired by the reviewer.

**Reviewer #2:**

Some critical issues:

1) The rationale for combinations of transporters deleted in 11, 12, and 13 deletion strains is not clear. The authors seemingly deleted random combinations of BAP3, BAP2, TAT1, TAT2, and AGP3. Why did the authors not pursue a more systematic or comprehensive deletion set? Why was BAP3 the only 11-deletion strain created and why not make all 5 11-deletion strains, especially considering the BAP3 11-deletion strain had a potential growth defect relative to 22Δ10α? The authors should clarify this rationale.

Response: We understand that, as described, our work seems to lack logic. Our initial goal was to delete 5 more amino acid permeases from 22∆10α, namely Bap2, Bap3, Tat1, Tat2 and Agp3, the remaining major amino acid permeases (using the data published in Regenberg, 1999). We started to delete in parallel *BAP3* and *BAP2-TAT1*, but only the former was successful at the first attempt, leading to 22∆11. We then used this strain to delete in parallel the other genes, leading to 22∆12, 22∆12’, 22∆13. As mentioned in the text (lines 345-347), the slow growth and the fact that further deletions did not lower much the background of 22∆10α, we did not attempt more deletions. It was much later that we found that some 22∆11 siblings did not display this slow growth phenotype, and, because of time and funding constraints, we could not repeat the deletion process from one of those siblings.

2) The authors claim the new strains will be useful for future studies of amino acid transport, yet the new strains did not exhibit significantly altered amino acid transport properties compared to the parental strain. What is the advantage of using the new strains over the classical and now sequenced 22Δ10α strain?

Response: We are now discussing this point in the new discussion (lines 361-367). Hopefully, it now addresses the reviewer’s comment.

3) The study notes inconsistencies between growth assays and radiolabeled amino acid uptake. However, this finding seems unresolved. The authors should provide more speculation for this finding or provide additional assays to clarify this discrepancy.

Response: We have included ample speculation in the discussion (lines 395-431). Hopefully, this addresses the reviewer’s comment.

Minor issues:

4) Page 12, Lines 232-235 – This sentence is very confusing. Why would deleting more amino acid transporters improve the uptake of amino acids?

Response: this sentence has been deleted, as part of the separation of the discussion from the results.

5) Page 14, Lines 266-268 – Is it actually 48 contigs across 16 scaffolds?

Response: Our assembly led to 25 contigs, which were assembled in 48 scaffold, as now explained in the text: “Using 8.12 Gbp of long-read PacBio sequencing data, the 22∆10α genome was assembled de novo using Canu to an estimated depth of 600x [31], leading to 25 contigs. Aligning the contigs to the S288C reference genome showed coverage over all chromosomes further supporting the assembly (Fig 4a). Furthermore, after reference-guided separation of erroneously collapsed contigs, scaffolding and polishing, no large duplications or translocations were identified (Fig 4b), leading to a 12.87 Mbp assembly in 48 scaffolds. Noticeably, 95% of the assembly was contained within the largest 16 scaffolds (12.2 Mbp, corresponding to the entire yeast genome), with the remaining 5% corresponding to totally ordered contigs not assembled into scaffolds, and aligning with miscellaneous regions of the genome (S3 Fig).”

6) Table 2 is confusing overall. Please clearly specify if all of the gene deletions/inactivations are in agreement with the expected mutations. Consider adding a column for observed deletion length. Were any deletions/inactivations different from what was expected? Were any adjacent genes or control elements affected? Would be helpful to stick to either bp or kbp for all lengths.

Response: Thank you for this suggestion. We have revised Table 2, providing more details about the expected (when known) and observed mutations. We also modified the corresponding text in the results, clarifying that the nature of most of the mutations were not known – only that of the mutations in GNP1 and AGP1, created by our lab, were known (L274-276).

**Responses - Figure 1: Comparison of the uploaded figure file and the one obtained after conversion to pdf by the PlosONE website.**

**Responses - Figure 2: Original scan the corresponding picture in S2 Fig.**
